# Genito-Urinary and Syphilitic Diseases

**Published:** 1898-10

**Authors:** Byron H. Daggett

**Affiliations:** Buffalo, N. Y.


### GENITO-URINARY AND SYPHILITIC DISEASES.

Conducted by BYRON H. DAGGETT, M. D., Buffalo, N. Y.

### MOVABLE KIDNEY AND ITS TREATMENT.

BIDWELL (*Lancet*, April 16, 1898,) says there are two forms of movable kidney—the "floating" and "dislocated." The symptoms vary greatly and are often most marked when the displacement is least noted. They are a dragging pain in the loins, sometimes simulating renal colic; digestive disturbances, pain, vomiting, hepatic colic, "gastric crisis" and neuralgia. Einhorn alleges that many of the symptoms are due to concomitant conditions, which usually follow or precede renal ptosis.

However, some of these symptoms may be traced directly to the movable kidney, such as result from the pressure of tumor, dragging on adhesions to the duodenum, or the renal plexus and urinary obstruction. The apparent enlargement of the displaced kidney is due to its covering of abdominal parieties. The examination should be made while the patient is postured in various ways from the upright to the horizontal.

Kendal Franks asserts that the anatomical relation and connection of the kidney afford the most rational explanation of the condition. The surface of the kidney is marked by the impression of the organs in its immediate environment. These organs are the liver, the ascending colon and spleen. Some pathologic condition may be the predisposing and trauma be the determining cause of dislocation.

Ptosis of the liver causes gastric crises, hydronephrosis and atrophy of the kidney, constipation and constant pain and kinking of the bowel. Severe symptoms call for active treatment and the question of operative interference must be considered.

Nephrectomy and nephrorraphy or nephropexy: Nephrectomy has been superseded (except in cases of pathologic destruction of the kidney substance) by nephropexy. Some of the methods proposed for anchoring the kidney are: (1) The kidney is exposed and the perirenal fat is abraded; (2) The kidney is attached to the lumbar-wound by stitches through its capsule; (3) The capsule is attached by sutures to the lumbar wound; (4) The sutures are passed through the kidney substance; (5) The kidney is sutured to the twelfth rib; (6) Vulliet's method by fixing the kidney with a tendonous strand from the longissimus dorsi muscle. R. H. Reed proposed transfixing the kidney, reaching it through the abdominal opening.

Vulliet's method is done as follows: An incision four inches in length is made just below and parallel to the twelfth rib, exposing the kidney, which is freed from its connections, another incision, two inches long, is made parallel and next to the spine, beginning opposite the first lumbar vertebra and exposing the tendon of the longissimus dorsi muscle, a thread of this tendon, about a sixth of an inch wide, is detached with a scalpel and a finger introduced underneath it. On pulling, a strip eight or nine inches long is detached from the rib, leaving the lower end attached to the spine of the first lumbar vertebra. Incisions are made in the upper and lower ends of the capsule of the kidney. Pass a probe threaded with this strip of tendon from the dorsal into the lumbar wound and thence through these incisions from below and then out through the dorsal opening. Pull this strip tense and it draws and anchors the kidney up under the ribs. Attach the free end of this string to the muscle, close the wound without drainage. In the lumbar wound use deep buried sutures and close both wounds with catgut sutures.

Bidwell reports three successful cases operated upon in this way. This living suture is unirritating, not quickly absorbed and the peritoneal cavity is not opened. "With Vulliet's method I feel that one can offer every prospect of permanent relief and so I should urge the operation even in preference to the application of a belt, since its results are much more satisfactory and the procedure is practically devoid of risk."

---

### THE SURGERY OF THE KIDNEY.

HENRY MORRIS (*Lancet*, April 16, 1898,) states that the practicability of nephrotomy was repeatedly discussed from the sixteenth to eighteenth centuries, but surgical treatment of the kidney was not employed systematically until the second half of the present century.

Simon, of Heidelberg, successfully removed a kidney for the cure of a ureteral fistula following ovariotomy, August 2, 1869. He did this regardless of the fact that Royer affirmed that it was "madness to dream of extirpating a kidney in the human subject." Previous to this time it had been demonstrated many times that animals could live with only one kidney. About a year afterward Gilmore, an American, successfully performed a nephrectomy, removing a diseased kidney from a woman five months pregnant, without producing a miscarriage.

A. E. Dunham did the first nephrectomy in England, May 14, 1872, and during the following decade several operators reported successful ablations of the kidney. It was ten years after Simon's successful nephrectomy was performed before the first nephrectomy was done in France. Nephrotomy never was systematically practised until 1870; previous to this time fluid swellings and perinephritic abscesses were opened by thermocautery or incision.

Annandale in 1869, and Spencer Wells in 1870, wrote in favor of nephrectomy, in some cases of renal calculus. During the ten years from 1870-1880 nephrotomy was done in a half dozen cases for the extraction of calculi and for pyonephrosis. Morris in 1880 removed by lumbar incision a large calculus from the kidney of a young woman and demonstrated that the kidney could be safely and freely incised. Dr. Morris reported thirty-four nephrotomies with only one death.

In 1882, Hahn, in Berlin, introduced an operation at first called nephrorraphy and afterward named nephropexy by the French. The purpose was to fix and anchor movable kidneys. In this operation the incision is made along the sacrolumbar muscle, from the twelfth rib to the crest of the ileum and through the quadratus lumborum. The capsule of the kidney is incised along the outer border and separated to some extent from that organ. The margins of the capsule are dragged into and stitched to the abdominal wound. Nephropexy is an important advance since it superseded nephrectomy for movable kidney.

A preferable method of attaching the kidney is fixation by means of deep sutures through its body and then passed into the cut edges of the transversalis fascia and the aponeurosis of the transversalis muscle. Tuffier recommended partial decortication of the kidney and suturing the tunica propria to the edges of the parietal wound. Vulliet, of Geneva, strips out a long, slender slip of the tendon of the erector spinæ and uses this as a means of fixation. Successful nephrolithotomy demonstrated that the kidney could be freely incised without producing fatal hemorrhage and that it would readily heal, then followed another advance, viz.: excision of the diseased portions of this organ. Numerous cases of partial excision of the kidney are reported and on record.

